# Epstein-Barr Virus Reactivation after Infliximab in Rheumatoid Arthritis: A Case Report

**DOI:** 10.1155/2011/530568

**Published:** 2011-06-30

**Authors:** Michele Colaci, Marco Sebastiani, Gilda Sandri, Marisa Meacci, Clodoveo Ferri

**Affiliations:** ^1^Rheumatology Unit, University of Modena and Reggio Emilia, Policlinico di Modena, Via del Pozzo 71, 41100 Modena, Italy; ^2^Microbiology and Virology Service, Policlinico di Modena, Via del Pozzo 71, 41100 Modena, Italy

## Abstract

TNF-alpha blockers represent one of the most important therapeutic strategies for rheumatoid arthritis, but their use has raised the question about their safety profile, particularly in respect to viral infections/reactivations. We describe the case of a patient who developed a symptomatic EBV reactivation 11 days after the first infusion of infliximab.

## 1. Introduction

Rheumatoid arthritis (RA) is a chronic disease characterized by inflammation of the synovial tissue of the diarthrodial joints, possibly leading to bone erosion and articular cartilage destruction. 

The pathogenesis of RA is based on an abnormal immune response, in which some cytokines, such as tumor necrosis factor- (TNF-) alpha, interleukin 1 and 6, play a pivotal role. Therefore, the development and the use of biologic agents, mainly the TNF-alpha blockers, represents one of the most important therapeutic strategies to treat RA patients nonresponder to traditional disease-modifying agents (DMARDs) [1].

In fact, TNF-alpha blockers have unequivocally demonstrated their efficacy in reducing disease activity and in decreasing the probability of articular damage, especially in association to methotrexate [1]. The widespread use of TNF-alpha blockers has raised the question about their safety profile; in fact, it has been well recognized that these biologic agents interfere with a number of immune defense pathways, particularly those correlated with the response against intracellular microbes [2].

The data from clinical trials and from safety registries have identified an increased risk for certain infections, first of all Tuberculosis [3]. Moreover, the risk of reactivation of certain viruses, including Epstein-Barr virus (EBV), has been suspected in patients treated with TNF-alpha blockers, and it has been clearly shown in transplant recipients and other immunosuppressed subjects [4]. Recently, a few studies have been published in order to investigate this hypothesis, concluding that TNF-alpha blockers have only a minimal, if any, influence on latent viral replication [5–9].

Here, a case of symptomatic EBV reactivation in a RA patient treated with infliximab is described.

## 2. Case Report

B.M., a 20-years-old female was referred to our Rheumatologic Clinic in March 2009 for an overt arthritis involving the third proximal interphalangeal (PIP) joint of the left hand and the first metacarpophalangeal (MCP) joint of the right hand. Beside arthritis, which appeared six months before, the patient presented fatigue, morning joint stiffness for about three hours, and more recently pain also in the wrists and in the other MCP and PIP joints. At the physical examination, both the knees and wrists were swollen, together with the firstly involved joints. Laboratory tests showed slight increase of erythrocyte sedimentation rate (24 mmh) and c-reactive protein 1.4 mg/dL, presence of serum rheumatoid factor (182 UI/mL; n.v. < 14), anticitrullinated peptide antibodies (251.6 UI/mL; n.v. < 25), while antinuclear antibodies were negative. Firstly, in the hypothesis of reactive arthritis because of a transient genital infection by Ureaplasma urealyticum, prednisone 12.5 mg/day tapered to 5 mg was administered for about three months; but, arthritis did not show any significant improvement. Radiograms of the hands evidenced typical marginal erosion at the basis of the phalanx of the third PIP joint. On these basis, a diagnosis of early RA was made, and a treatment with methotrexate 10 mg/week plus prednisone 5 mg/day and diclofenac 150 mg/day was started.

**Figure 1:**
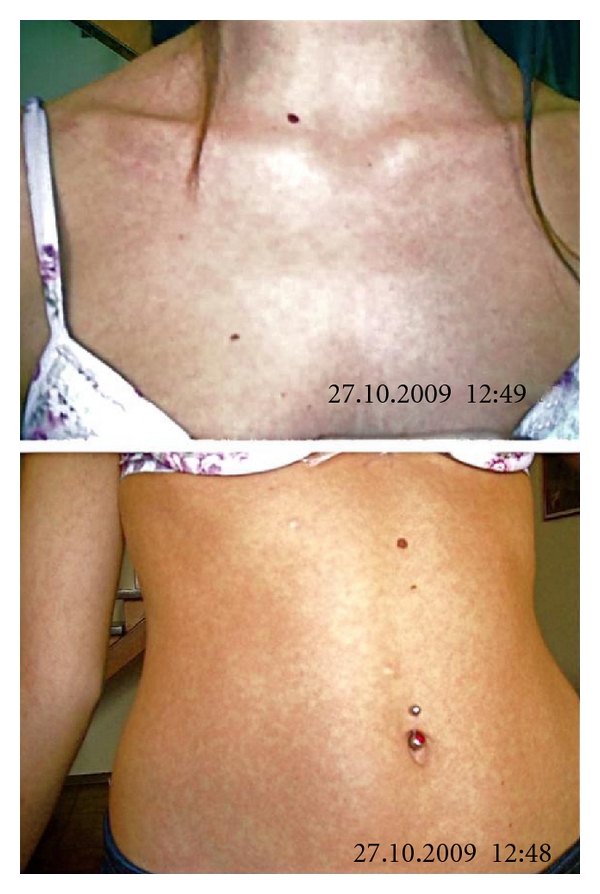
The rash present on the patient's trunk 11 days after the infliximab infusion.

In the following 3 months, the patient did not experience a significant improvement of her symptoms, so she continued to take diclofenac every day. Similarly, leflunomide 20 mg/day in association with diclofenac and steroids did not show satisfying results. After a negative screening for hepatitis B and C virus and Mycobacterium tuberculosis, a treatment with infliximab 3 mg/kg was decided, considering the activity and the severity of arthritis. The first infusion was performed on 15th October 2009, without steroid or antihistaminic premedication; no adverse reactions were registered during the infusion and in the following week. On 26th October, the patient presented a macular rash on the neck and the trunk (Figure 1), and painful lymphonodes behind the ears and on the neck; fever of 37.5°C and sore throat were also present.

Suspecting a viral infection, a serological evaluation for measles, EBV, Cytomegalovirus, and Parvovirus B19 was performed, using an immunoenzymatic reaction kit (Technogenetics-Bouty, Milan, Italy). EBV-VCA IgM resulted 2.01 (values > 1.10 were considered positive), EBV-VCA IgG 121 (values > 11.5 were considered positive), along with serum EBV-EBNA IgG (memory antibodies). The IgM antibodies directed against the other investigated viruses were negative. 

All the above symptoms disappeared in five days spontaneously and did not reappear up to date. In the same time, arthritis dramatically improved after the first infusion of infliximab, with apparent resolution of synovitis of the wrists, knees, and hands. The second infliximab infusion was done one month later, after the complete resolution of the EBV reactivation symptoms.

## 3. Discussion

The case here described represents a viral reactivation complicating TNF-alpha blocker treatment. EBV reactivation was clinically suspected because of the appearance of fever, macular rash, pharyngitis, and lymphoadenopathies. It was confirmed by the presence of EBV-VCA IgM antibodies in the serum, whereas the EBV-EBNA IgG antibodies demonstrated a previous contact with the virus. A previous positivity of EBV-VCA IgM cannot be definitely excluded; however, the patient's clinical picture is reasonably suggestive of EBV reactivation. 

Several studies have investigated the possibility of an EBV reactivation in patients treated with infliximab, showing no significant increased risk [5–8]. In a study of 60 patients with Crohn's disease treated with infliximab, none showed EBV viremia or any clinical manifestation of EBV infection after the treatment [6]. In addition, no significant modifications of EBV serological markers were demonstrated in a series of 68 RA patients treated with infliximab and 48 with etanercept [7]. Finally, serum EBV DNA was never found in a series of anti-EBV IgG-positive patients treated with TNF-alpha blockers [8], while the EBV viral load and the number of IFN*γ*-producing T cells after viral peptide stimulation were not significantly increased after TNF-alpha blockers in patients with RA or spondylarthropathy [9].

On the other hand, Komatsuda et al. described the case of a RA patient who developed a Hodgkin-like lymphoproliferative disorder, related to EBV, at the 30th month of infliximab treatment, which dramatically regressed after anti-TNF-alpha discontinuation [10]. Indeed, it has been observed that the immune response to EBV is slightly impaired in RA patients [11]; consequently, this possible inefficient control of the virus might be exalted by the treatment with TNF-alpha blockers in a few RA patients. 

It is presumable that the viral reactivation might be a rare event, which is difficult to verify in the relatively small series of patients previously investigated [5–9]. Moreover, it is also possible that this event may be misdiagnosed or entirely overlooked because it could be characterized by nonspecific, often mild clinical manifestations. In our case, the clinical picture was quite relevant to suspect the diagnosis, even though it was transient and substantially did not influence the infliximab schedule and therapeutic response. In general, we cannot exclude the fact that apparently benign EBV reactivation might trigger more serious complications due to the potential EBV-driven autoimmune-lymphoproliferative phenomena, particularly in genetically predisposed patients. Further studies could clarify if EBV serological evaluations should be included in the screening before anti-TNF-alpha therapy.
